# Supplementary material for: Beauty Beware: Associations between Perceptions of Harm and Safer Hair-Product-Purchasing Behaviors in a Cross-Sectional Study of Adults Affiliated with a University in the Northeast
Source: Int J Environ Res Public Health. 2023 Nov 30;20(23):7129. doi: 10.3390/ijerph20237129 (PMC10706836; doi:10.3390/ijerph20237129)
Supplement: Supplementary file 1 [file ijerph-20-07129-s001.zip › ijerph-2709989-supplementary.pdf]

	Females who usually use a healthy product app or website when buying a hair product to use at home			Females who usually read the ingredients on the label when buying a hair product to use at home			Females who usually look for labels indicating the product is made with natural, non-toxic, or eco-friendly ingredients when buying a hair product to use at home		
**n/N (%)**	**34/478 (7.1)**			**197/485 (40.6)**			**189/484 (39.0)**		
**PERCEPTIONS**	**OR (95% CI)**	**Log OR (95% CI)**	***P***	**OR (95% CI)**	**Log OR (95% CI)**	***P***	**OR (95% CI)**	**Log OR (95% CI)**	***P***
Strongly agree that the personal care products I use affect my health.	2.29 (0.97, 5.39)	0.83 (–0.03, 1.69)	0.059	3.31 (1.90, 5.75)	1.20 (0.64, 1.75)	<0.001	3.36 (1.93, 5.84)	1.21 (0.66, 1.76)	<0.001
Strongly agree that consumers should be concerned about the health effects of personal care products.	4.14 (1.95, 8.80)	1.42 (0.67, 2.17)	<0.001	4.41 (2.84, 6.86)	1.48 (1.04, 1.93)	<0.001	4.34 (2.79, 6.75)	1.47 (1.03, 1.91)	<0.001
Strongly disagree that there is no reason to worry about the health effects from chemicals that might be in personal care products.	2.95 (1.38, 6.30)	1.08 (0.32, 1.84)	0.005	2.67 (1.78, 4.00)	0.98 (0.58, 1.38)	<0.001	2.70 (1.80, 4.04)	0.99 (0.59, 1.40)	<0.001
Strongly disagree that the benefits of using personal care products outweigh any risks from exposure to toxic chemicals that might be in these products.	2.07 (0.88, 4.85)	0.73 (–0.12, 1.58)	0.095	3.29 (1.92, 5.67)	1.19 (0.65, 1.73)	<0.001	3.33 (1.94, 5.74)	1.20 (0.66, 1.75)	<0.001
Strongly disagree that the Food and Drug Administration (FDA) and other governmental agencies do a good job of regulating personal care products to ensure they are safe for consumers.	3.18 (1.40, 7.22)	1.16 (0.34, 1.98)	0.006	1.56 (0.95, 2.85)	0.45 (–0.05, 0.95)	0.080	1.57 (0.95, 2.60)	0.45 (–0.05, 0.95)	0.076

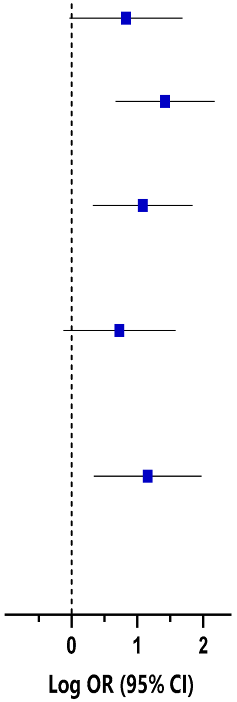

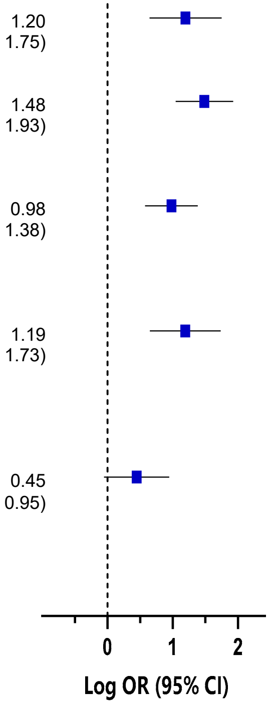

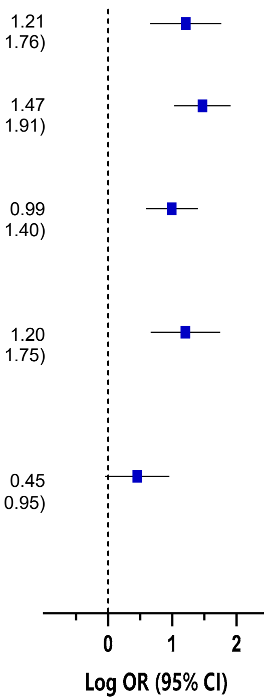

Supplementary Figure S1. Adjusted associations between perceptions of harm and hair product purchasing behaviors among females in the study. Associations between perceptions of harm (strongly agree/strongly disagree to a perception vs. all other responses) and hair care purchasing behaviors among females were examined using multivariable-adjusted logistic regression models. These associations were reported as odds ratios, and the corresponding log odds ratios were displayed in plots (shown above), which visualized the log odds of always/usually exhibiting "safer" purchasing behaviors versus the log odds of not always/usually exhibiting "safer" purchasing behaviors, while controlling for age (continuous), income, marital status, and race, and ethnicity.

Females who use ≥3 hair products on average during a 24–48-hour period

n/N (%) **150/485 (31)**

**PERCEPTIONS**	**OR (95% CI)**	**Log OR (95% CI)**	***P***
Strongly agree that the personal care products I use affect my health.	0.92 (0.52, 1.62)	–0.08 (–0.65, 0.48)	0.776
Strongly agree that consumers should be concerned about the health effects of personal care products.	1.02 (0.66, 1.57)	0.02 (–0.42, 0.45)	0.942
Strongly disagree that there is no reason to worry about the health effects from chemicals that might be in personal care products.	0.99 (0.65, 1.50)	–0.01 (–0.43, 0.41)	0.962
Strongly disagree that the benefits of using personal care products outweigh any risks from exposure to toxic chemicals that might be in these products.	0.80 (0.46, 1.40)	–0.22 (–0.79, 0.34)	0.433
Strongly disagree that the Food and Drug Administration (FDA) and other governmental agencies do a good job of regulating personal care products to ensure they are safe for consumers.	1.11 (0.67, 1.86)	0.11 (–0.41, 0.62)	0.686

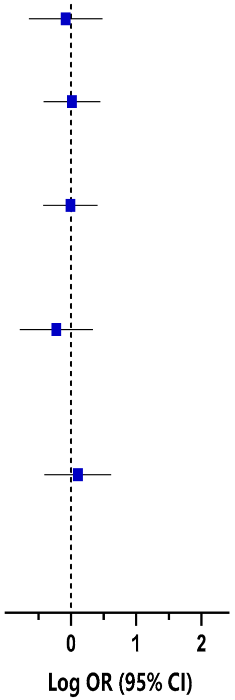

Supplementary Figure S2. Unadjusted associations between perceptions of harm and hair product usage behaviors among females in the study. Associations between perceptions of harm (strongly agree/strongly disagree to a perception vs. all other responses) and hair care use behaviors among females were examined using unadjusted logistic regression models. These associations were reported as odds ratios, and the corresponding log odds ratios were displayed in a plot (shown above), which visualized the log odds of always/usually exhibiting "safer" behaviors versus the log odds of not always/usually exhibiting "safer" behaviors around PCP hair product use, while controlling for age (continuous), income, marital status, and race and ethnicity.

PERCEPTIONS	Males who usually use a healthy product app or website when buying a hair product to use at home OR (95% CI)	Log OR (95% CI)	*P*	Males who usually read the ingredients on the label when buying a hair product to use at home OR (95% CI)	Log OR (95% CI)	*P*	Males who usually look for labels indicating the product is made with natural, non-toxic, or eco-friendly ingredients when buying a hair product to use at home OR (95% CI)	Log OR (95% CI)	*P*
n/N (%)	5/79 (6.3)			20/82 (24.4)			22/80 (27.5)		
Strongly agree that the personal care products I use affect my health.	3.45 (0.32, 36.98)	0.62 (–0.57, 1.81)	0.306	10.00 (1.77, 56.67)	2.30 (0.57, 4.04)	0.009	4.07 (0.83, 19.95)	1.40 (–0.18, 2.99)	0.083
Strongly agree that consumers should be concerned about the health effects of personal care products.	4.82 (0.71, 32.85)	0.79 (–0.17, 1.75)	0.109	7.60 (2.12, 27.31)	2.03 (0.75, 3.31)	0.002	7.71 (2.03, 29.35)	2.04 (0.71, 3.38)	0.003
Strongly disagree that there is no reason to worry about the health effects from chemicals that might be in personal care products.	8.59 (1.28, 57.46)	1.08 (0.13, 2.03)	0.027	9.33 (2.77, 31.46)	2.23 (1.02, 3.45)	<0.001	13.50 (3.62, 50.29)	2.60 (1.29, 3.92)	<0.001
Strongly disagree that the benefits of using personal care products outweigh any risks from exposure to toxic chemicals that might be in these products.	12.38 (1.79, 85.58)	1.26 (0.29, 2.22)	0.011	6.14 (1.68, 22.44)	1.81 (0.52, 3.11)	0.006	4.95 (1.37, 17.84)	1.60 (0.32, 2.88)	0.015
Strongly disagree that the Food and Drug Administration (FDA) and other governmental agencies do a good job of regulating personal care products to ensure they are safe for consumers.	3.45 (0.32, 36.98)	0.62 (–0.57, 1.81)	0.306	1.27 (0.23, 7.10)	0.24 (–1.49, 1.96)	0.788	1.35 (0.23, 7.95)	0.30 (–1.47, 2.07)	0.740

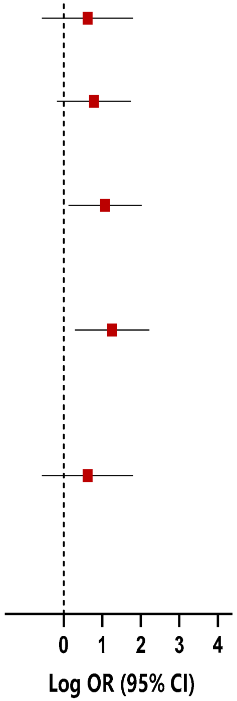

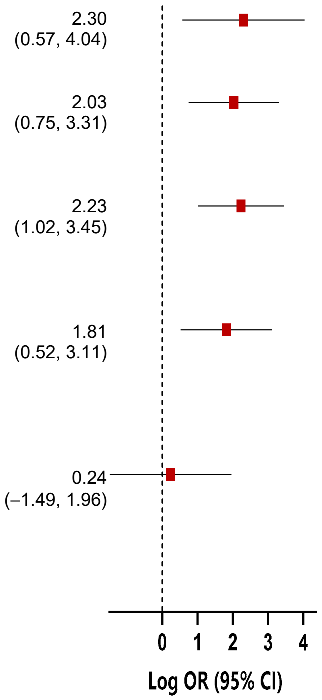

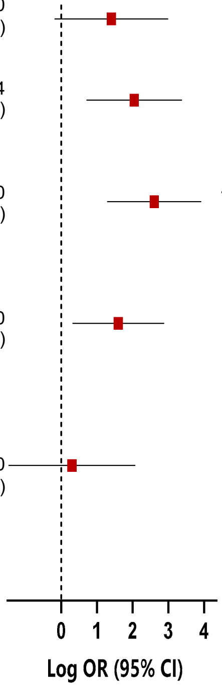

Supplementary Figure S3. Adjusted associations between perceptions of harm and hair product purchasing behaviors among males in the study. Associations between perceptions of harm (strongly agree/strongly disagree to a perception vs. all other responses) and hair care purchasing behaviors among males were examined using multivariable-adjusted logistic regression models. These associations were reported as odds ratios, and the corresponding log odds ratios were displayed in plots (shown above), which visualized the log odds of always/usually exhibiting "safer" purchasing behaviors versus the log odds of not always/usually exhibiting "safer" purchasing behaviors, while controlling for age (continuous), income, marital status, and race and ethnicity.
